# Coronavirus disease 2019: Global Contamination and Global Cleanup

**DOI:** 10.31661/gmj.v9i0.1921

**Published:** 2020-09-11

**Authors:** Amir Norouzpour, Mahmood Nejabat, Alireza Mehdizadeh

**Affiliations:** ^1^Shiraz University of Medical Sciences, Shiraz, Iran

**Keywords:** COVID, Coronaviruse, Habits, Innovation

## Dear Editor,

 T

he Coronavirus disease 2019 (COVID-19) is a global health concern that involved more than 12 million people around the world [1]. The origin of the COVID-19 pandemic is still obscure. However, whether it is a product of laboratory-based manipulation or a result of the zoonotic transfer, we as humans living together on the Earth need to reexamine our thoughts and behaviors. A tradition or culture like a food culture might pose hazards not only for people who developed it but also for the world globally. We do not usually contemplate our culture-driven, or society-driven, behaviors until they lead to a catastrophe. That is, of course, too late. We need scientific research to weigh up the advantages and disadvantages of our habits. Scientific methods enable us to explore alternative ways of which the benefits outweigh the risks to our health. We would be able to adopt a healthier lifestyle. Our decisions not only change our lives but also influence all living and non-living beings relatively.

 Moreover, scientific innovations spread globally and affect many lives. Therefore, an innovative idea should be theoretically discussed before performing an action based on innovation. Scientific forums for sharing and exchanging ideas and hypotheses should be created and extended. The benefits and costs should be discussed, and the effects on our health and lives should be predicted as possible. The innovations must have the least adverse effects on wildlife and be consistent with ecosystem health. Our science should be cleaned up from ugly innovations. We are responsible for our innovations. Also, international medical and health organizations should follow the policy on exploring and minimizing public health hazards. We need to feel that we are living together, not against each other on the Earth.

The warning signals in the scientific literature also help us to improve our lifestyles. Chinese scientists warned us a year ago that a Coronavirus outbreak would re-emerge [2]. Whether it turns out to be true or not, we need to be more sensitive to the warning signals in the scientific papers in the future. The notified signals need to be evaluated using scientific methods. There should be enough evidence supporting that the signal has a crucial influence on our lives if neglected. Furthermore, scientists should make appropriate warning signals at the appropriate time; therefore, we need to be fully alert to the possibility of a health disaster. Convincing evidence should be provided for our claims, and there should be scientific forums for debates on forthcoming health issues. It would have been better to contribute politicians to the debates on public health disasters. Science and politics should be connected more closely. The scientific warning signals alert the community to public health disasters, and politicians’ decisions help to prevent disasters happening. The signals eventually inspire us to change our lifestyle and improve the health-related quality of our lives worldwide.

 Now, scientists are attempting to find prophylactic and therapeutic interventions for COVID-19. Their discovery will be used globally, saving lives all over the world. That is the power of science and the responsibility of scientists. Our thoughts and actions influence all lives globally; therefore, we should be reexamining our thoughts and behaviors.

## Conflict of Interest

 None.
